# Retraction Note to: Extranuclear ERα is associated with regression of T47D PKCα-overexpressing, tamoxifen-resistant breast cancer

**DOI:** 10.1186/s12943-017-0697-5

**Published:** 2017-07-17

**Authors:** Bethany Perez White, Mary Ellen Molloy, Huiping Zhao, Yiyun Zhang, Debra A. Tonetti

**Affiliations:** 1 Department of Biopharmaceutical Sciences, College of Pharmacy, University of Illinois at Chicago, 833 S. Wood Street, Chicago, IL 60611 USA; 2 Current address: Department of Medicine, Harvard Medical School, Boston, MA 02115 USA

## Retraction Note

This article [1] has been retracted by the authors after they discovered an error in the data. The authors recently performed Short Tandem Repeat (STR) analysis on all of their cell lines and discovered that the cell clone T47D:A18/PKCα is in fact MCF-7/PKCα. They have verified this using next generation sequencing of the p53 mutation that is a hallmark of T47D cells. All other cell lines reported in this article are as originally described. As a result of this error, parts of the article are inaccurate which affects the article’s conclusions. The authors will be given the opportunity to resubmit their corrected data.
